# Retroperitoneal Dumbbell-Shaped Lipoma: Laparoscopy-Assisted Hybrid Surgical Approach

**DOI:** 10.7759/cureus.90564

**Published:** 2025-08-20

**Authors:** Haridev Sankar, Naveen Alexander, K Arun Kumar

**Affiliations:** 1 General Surgery, Sri Ramachandra Institute of Higher Education and Research, Chennai, IND

**Keywords:** dumbbell tumor, femoral canal, groin mass, hybrid approach, laparoscopy-assisted excision, retroperitoneal lipoma

## Abstract

Retroperitoneal lipomas are benign tumors but are rarely seen extending into the femoral canal. A 43-year-old woman presented with a painless, progressively enlarging swelling in her right upper thigh. Imaging revealed a well-defined, fat-density, dumbbell-shaped mass measuring 17 cm, arising from the retroperitoneum and extending into the anterior thigh through the femoral canal. A trucut biopsy confirmed a lipoma. She underwent a successful laparoscopy-assisted open excision. Laparoscopic dissection facilitated mobilization of the intra-abdominal component, while a small groin incision allowed removal of the mass through the femoral canal. Histopathology confirmed a benign lipoma. As no defects were identified in the inguinal or femoral regions, mesh reinforcement was not required. Lipomas are common in subcutaneous tissue, but retroperitoneal lipomas extending through the femoral canal are rare and may mimic incarcerated hernias, complicating diagnosis. While open excision remains the standard treatment, the hybrid laparoscopic approach in this case provided a less invasive, precise, and safe method for complete tumor removal, resulting in an uneventful recovery. This case highlights a rare retroperitoneal dumbbell-shaped lipoma passing through the femoral canal and demonstrates the feasibility and advantages of a laparoscopy-assisted hybrid surgical approach.

## Introduction

Lipomas are the most common benign soft tissue tumors. They usually develop in subcutaneous tissues but are rarely found in the retroperitoneum. Primary retroperitoneal tumors themselves are uncommon, with an estimated incidence of 0.2-0.5 per 100,000 population per year, and retroperitoneal lipomas account for fewer than 2% of these tumors [1]. They are often asymptomatic until they reach a size large enough to compress adjacent structures. A dumbbell-shaped tumor, defined as two large lobes connected by a narrower neck that passes through a natural anatomical opening such as the femoral canal, is extremely rare and may mimic more common groin conditions, including femoral hernias or soft tissue sarcomas [2].

The femoral canal, bordered by the femoral vein on one side and the lacunar ligament on the other, is a frequent site for hernias, particularly in women. When a lipoma extends into this space, it may present as a non-reducible groin or upper thigh mass, making diagnosis challenging [3]. Imaging modalities such as contrast-enhanced CT and MRI are essential to confirm the fatty nature of the lesion, assess its extent, and rule out liposarcoma [4].

Surgical excision remains the standard treatment, both to establish a definitive diagnosis and to prevent complications. While traditional approaches involve open surgery through inguinal or abdomino-inguinal incisions, minimally invasive techniques are increasingly being used in selected cases [5]. This case describes a rare dumbbell-shaped retroperitoneal lipoma herniating through the femoral canal and highlights the successful application of a laparoscopy-assisted hybrid surgical technique.

## Case presentation

A 43-year-old woman, who had been asymptomatic three months earlier, presented with a swelling in her right thigh that had gradually increased in size over the past three months. The swelling had an insidious onset, was progressive, non-reducible, and painless. She reported no fever or other systemic symptoms. Bowel and bladder habits were normal, and her menstrual cycles were regular.

She was a mother of two, both delivered vaginally without complications. She had no significant past medical or surgical history, and her family history was unremarkable.

On general examination, her vital signs were stable. There was no pallor, jaundice, cyanosis, clubbing, lymphadenopathy, or pedal edema. The abdomen moved symmetrically with respiration, with no visible peristalsis, scars, or sinuses. On palpation, the abdomen was soft and non-tender, with no organomegaly. A digital rectal examination was normal.

**Figure 1:**
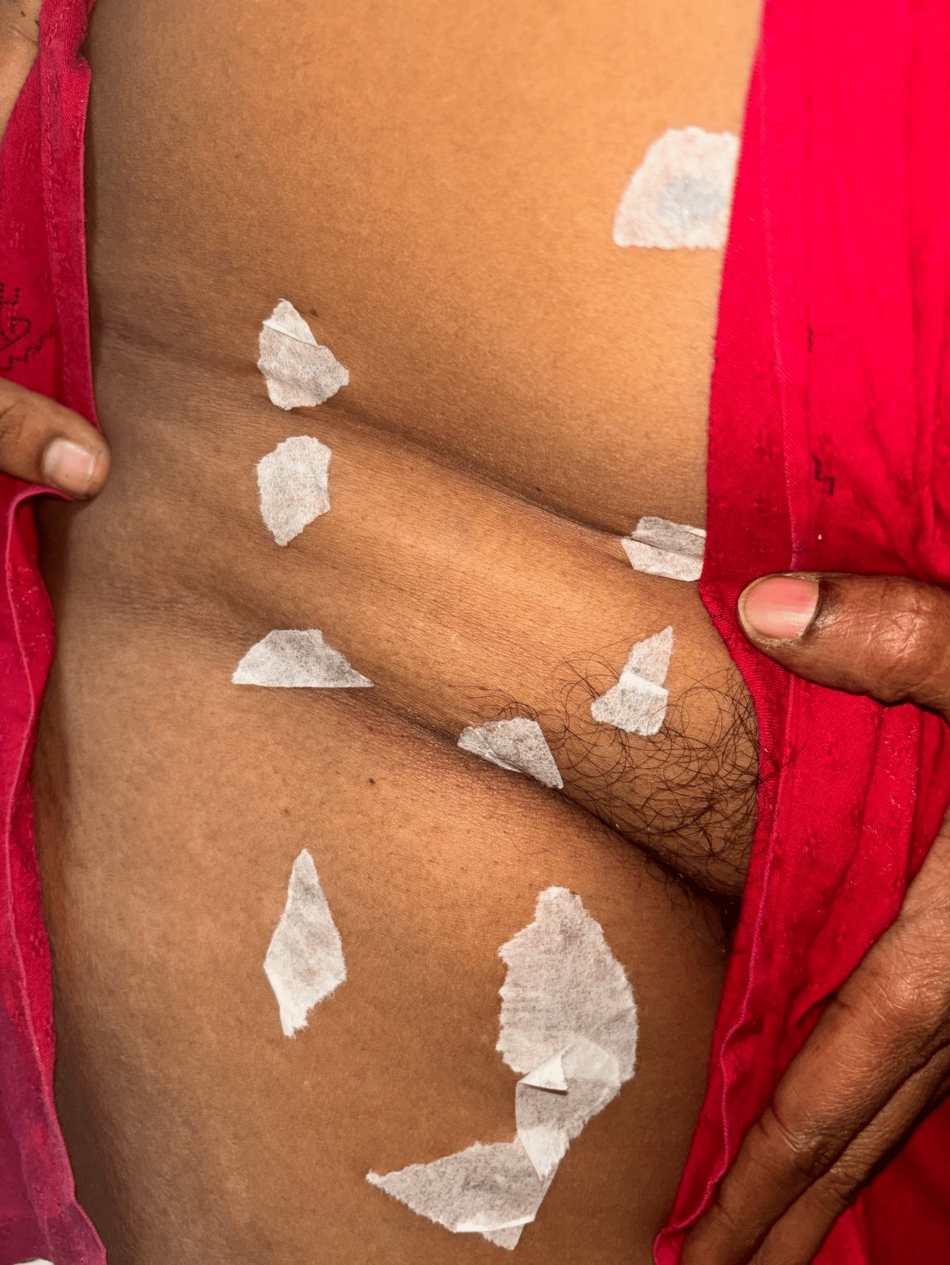
Surface marking of the retroperitoneal lipoma in the right thigh

Local examination of the right thigh revealed a firm, non-reducible swelling measuring 7 × 4 cm, located below and lateral to the pubic tubercle. The swelling had a lobulated surface, well-defined margins, was soft in consistency, and demonstrated a positive slip sign (slipping under the palpating fingers on examination) (Figure 1). Vaginal examination revealed no abnormalities.

**Figure 2:**
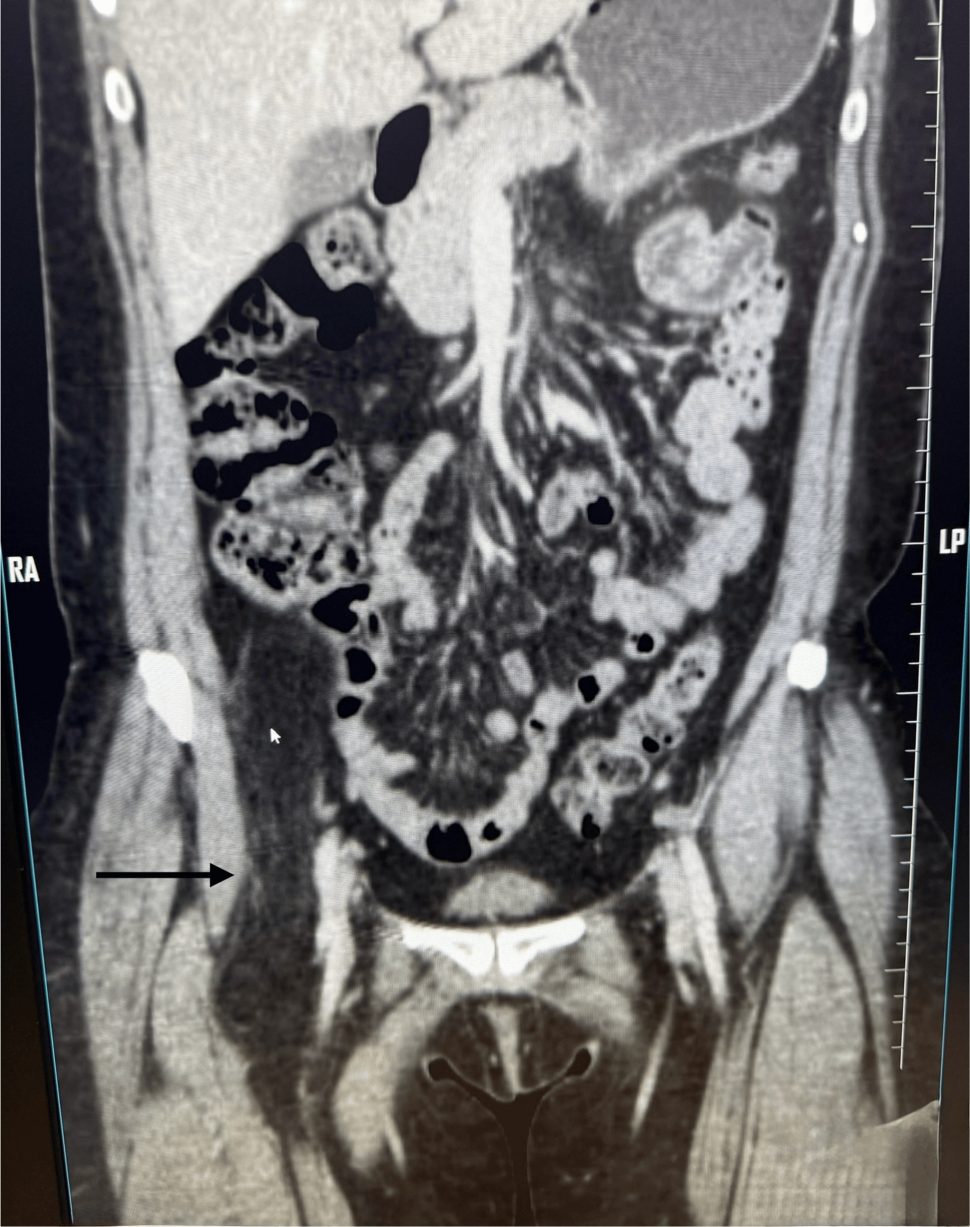
CECT scan of the abdomen showing a retroperitoneal lipoma (black arrow) extending through the femoral canal into the thigh CECT: contrast-enhanced computed tomography.

A contrast-enhanced computed tomography (CECT) scan of the abdomen and pelvis revealed a well-defined, fat-density, dumbbell-shaped mass in the right paracolic gutter extending into the right thigh through the femoral canal. The lesion measured 4.7 × 4.1 × 17 cm and was located adjacent to the right iliacus, near the cecum and ascending colon, and anterior to the right psoas muscle, with preserved fat planes in all directions. In the thigh, the mass displaced the femoral vessels outward (Figure 2).

An ultrasound-guided trucut biopsy of the thigh portion of the mass confirmed it was a benign lipoma.

**Figure 3:**
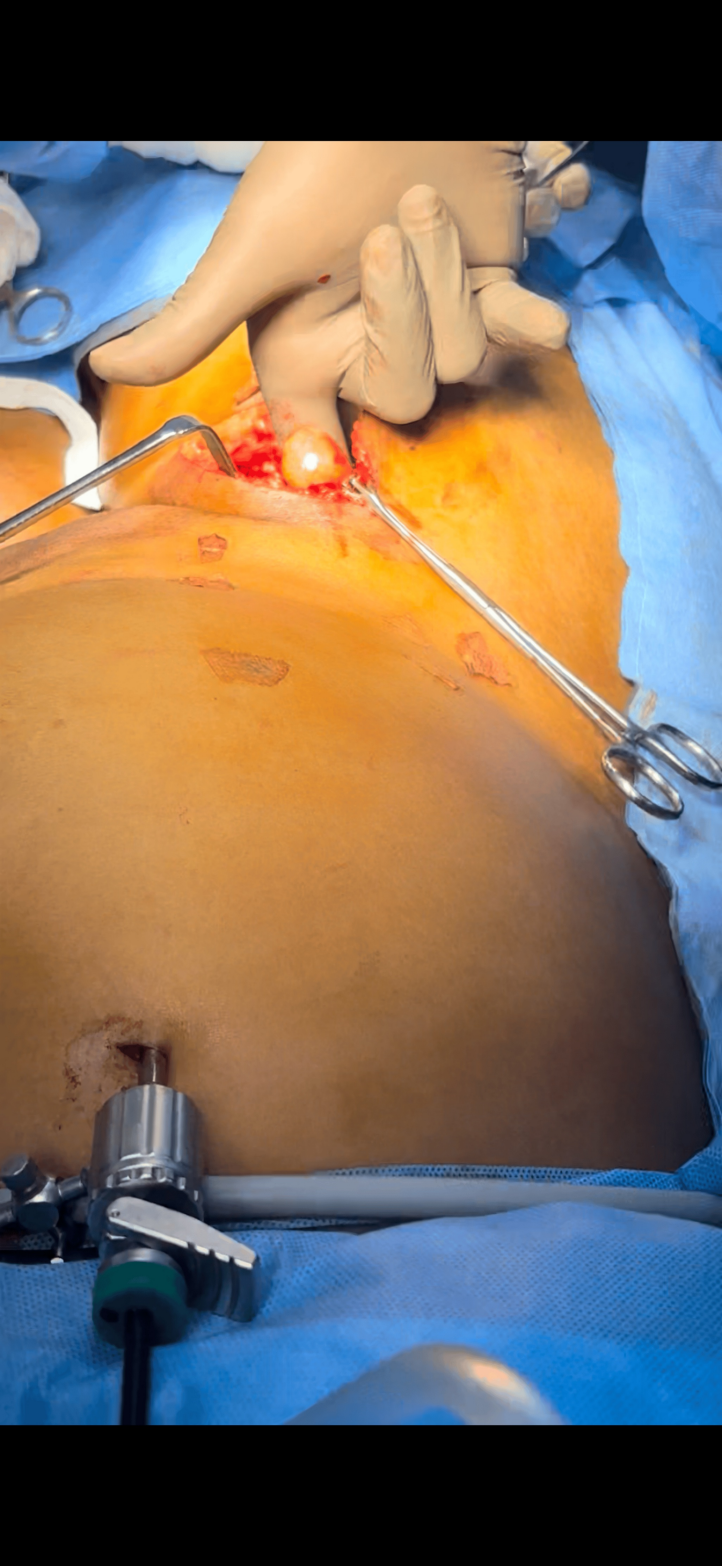
Specimen retrieval through the groin incision after laparoscopic mobilization

**Figure 4:**
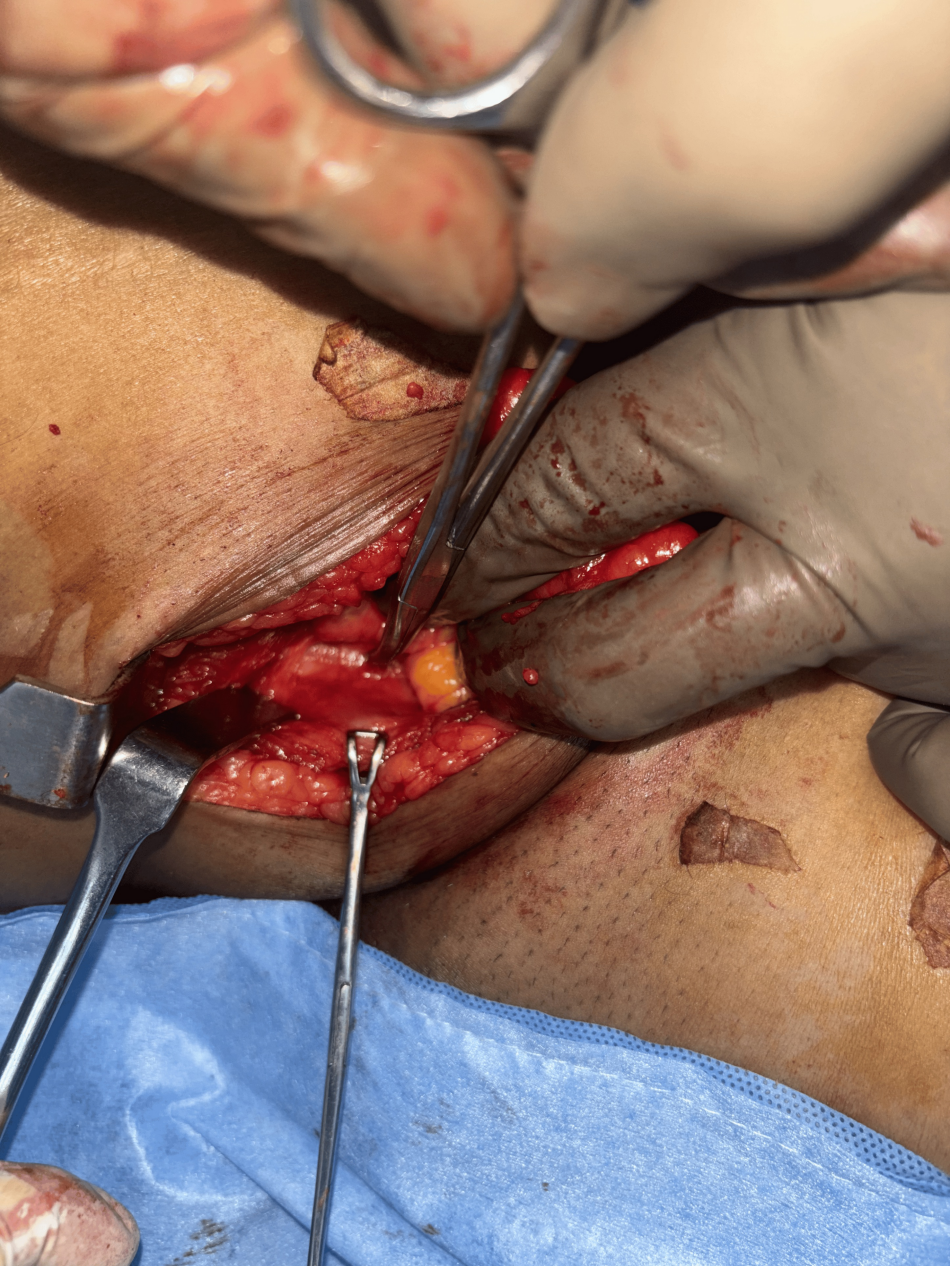
Intraoperative image showing the femoral vessels displaced laterally

**Figure 5:**
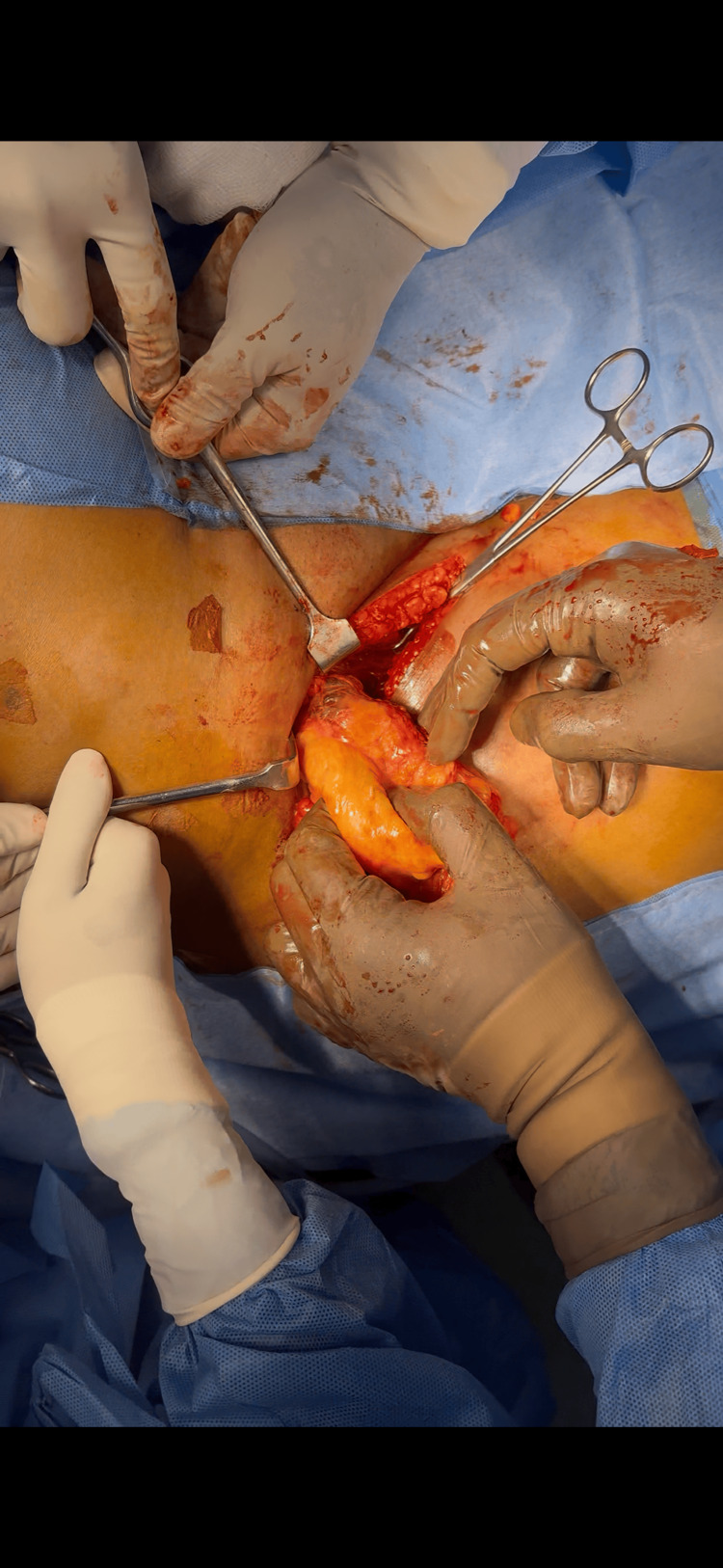
Specimen extraction

The patient underwent a laparoscopy-assisted open excision of the retroperitoneal lipoma. She was positioned supine, with arms adducted and chest and thigh strapped. A 10 mm port was placed above the umbilicus using the open (Hasson) technique for the camera and insufflation. Pneumoperitoneum was maintained at 14 mmHg with a flow rate of 10 L/min. Two 5 mm working ports were inserted along the right and left upper abdominal midclavicular lines. A peritoneal flap was created in the right lower abdomen to access the retroperitoneum. The mass was mobilized from adjacent structures: the psoas muscle posteriorly, the ureter inferiorly (preserved), and the iliac vessels medially (preserved). A 5 cm groin incision was then made about 2 cm below the inguinal ligament (Figure 3). Dissection through tissue layers exposed the lipoma, which was seen extending through the femoral canal. The femoral artery and vein were identified and preserved (Figure 4). The tumor was delivered through the groin incision (Figure 5). Laparoscopic inspection showed no inguinal or femoral defects; therefore, mesh reinforcement was not required.

**Figure 6:**
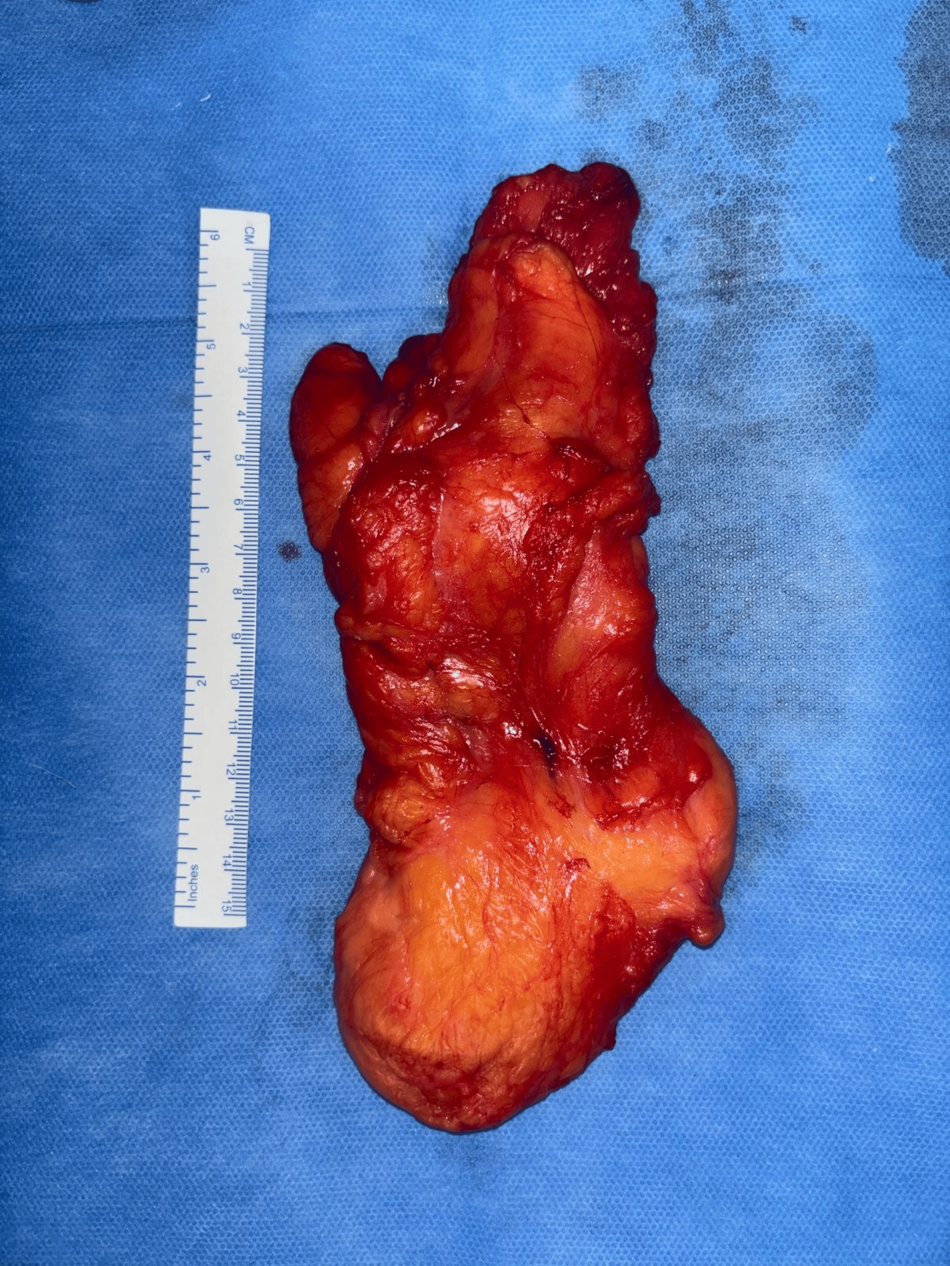
Excised specimen

**Figure 7:**
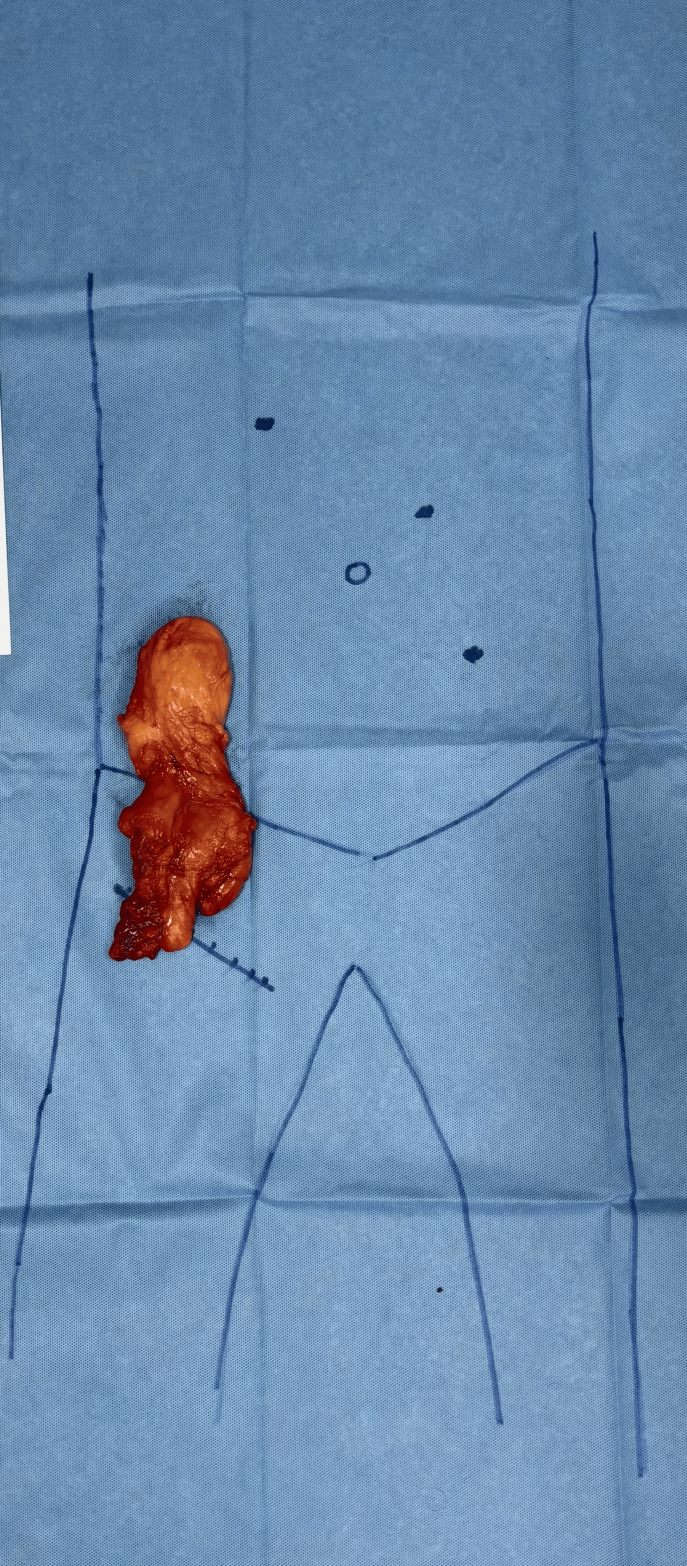
Excised specimen with marking

The peritoneal flap was closed using 2-0 absorbable barbed sutures. The abdominal port sites were closed with 2-0 nylon sutures, and the groin incision was closed in layers with 2-0 absorbable sutures for the subcutaneous tissue and 2-0 nylon for the skin (Figure 6 and Figure 7). The total operative time was 90 minutes, with an estimated blood loss of approximately 50 mL. The excised specimen measured 17 × 5 × 5 cm and weighed 150 g.

Histopathological analysis confirmed the mass was a benign lipoma. The patient recovered well after surgery and has remained symptom-free at her three-month follow-up. Clinical examination at that time was also normal. She has been advised to return for a six-month follow-up, which is awaited.

## Discussion

Lipomas are common benign tumors composed of mature fat cells. They most often develop in the subcutaneous tissue of the trunk, limbs, or neck. Primary retroperitoneal tumors are rare, with an estimated incidence of 0.2-0.5 per 100,000 population per year. Retroperitoneal lipomas are even less common, accounting for fewer than 2% of all primary retroperitoneal tumors [1]. A particularly unusual presentation occurs when a retroperitoneal lipoma extends through the femoral canal into the anterior thigh, where it can resemble a femoral hernia or a soft tissue tumor. This atypical appearance may delay diagnosis and complicate treatment.

The femoral canal is a narrow anatomical space bordered by the lacunar ligament medially and the femoral vein laterally. It is a common site for femoral hernias, especially in women, but it is extremely rare for a benign tumor such as a lipoma to pass through this region [6]. In such cases, the swelling is often firm, non-tender, and irreducible, mimicking an incarcerated hernia or a deep soft tissue mass. The absence of systemic symptoms, recent trauma, or prior surgery may further obscure the clinical diagnosis.

Imaging plays a crucial role in diagnosis, with CECT or MRI being the preferred modalities. Lipomas typically appear as well-defined, homogeneous fat-density lesions with preserved surrounding fat planes and no enhancement [7]. In our case, CECT revealed a dumbbell-shaped lipomatous mass measuring 17 cm, originating in the retroperitoneum and extending into the right thigh through the femoral canal, displacing adjacent structures, including the femoral vessels.

Several similar cases have been reported, each managed with different approaches. Kotohata et al. described a dumbbell-shaped retroperitoneal lipoma, 20 cm in size, mimicking a femoral hernia, which was removed through an extensive 15 cm open incision [6]. Nardi et al. reported a 14 cm retroperitoneal lipoma extending into the thigh through the inguinal canal, excised via Karakousis’s abdomino-inguinal approach, emphasizing the need for wide access in deep-seated tumors [8]. Gungor et al. described a 30 cm lipoma originating in the thigh and extending superiorly into the inguinal canal and inferiorly into the subsartorial canal, which was removed using an open excision approach [9]. Teh et al. reported a 6 cm femoral canal lipoma compressing the femoral vein and causing leg swelling; it was excised through a direct open femoral incision. Dissection was challenging due to vascular adhesions and resulted in a femoral vein tear, which required primary repair [10].

Compared with the above open approaches [6,8,9,10], which typically required large incisions and extensive dissection for safe tumor removal, as well as longer recovery periods and greater postoperative discomfort, our hybrid laparoscopy-assisted approach offered several advantages. In our case, complete removal was achieved in approximately 90 minutes, comparable to open techniques, but with a smaller groin incision, reduced postoperative pain, and earlier mobilization. The patient was started on a normal diet and mobilized on postoperative day (POD) 1, and discharged on POD 2 without complications. While no recurrence was noted at the three-month follow-up, longer surveillance is required.

In contrast to traditional open techniques, our laparoscopy-assisted method combined the benefits of minimally invasive retroperitoneal dissection with the safety of a limited groin incision for specimen retrieval. The laparoscopic view allowed precise dissection of the lipoma from adjacent structures, including the iliacus, psoas, ascending colon, and major vessels, without vascular injury. The femoral artery and vein were clearly visualized and preserved. After mobilization, the tumor was removed through a small skin-crease incision in the groin, thereby avoiding wide open exposure.

Our patient had an uneventful recovery and remained symptom-free three months after surgery. No femoral or inguinal defect was identified intraoperatively, so mesh reinforcement was not required.

This hybrid approach is rarely reported in the management of such tumors. Nomura et al. reported laparoscopic removal of a 7 cm retroperitoneal liposarcoma, with no recurrence at 24-month follow-up; however, their case did not involve extension through the femoral canal [11]. Our technique provides a safe and minimally invasive option, particularly valuable for anatomically complex tumors that span different compartments.

Histopathological analysis confirmed a benign lipoma. Although lipomas generally grow slowly, deep or rapidly enlarging lesions should be evaluated to exclude well-differentiated liposarcoma, especially in the retroperitoneum [12]. Complete excision with clear margins remains the treatment of choice. At the three-month follow-up, our patient has remained symptom-free with no evidence of recurrence.

This case underscores the importance of considering retroperitoneal lipoma in the differential diagnosis of groin or thigh masses, particularly when imaging shows fat-density characteristics. It also highlights the feasibility and benefits of laparoscopy-assisted open excision as a safe, less invasive alternative to extensive open surgery in selected cases.

Contraindications and limitations

Hybrid laparoscopy-assisted excision may not be appropriate in cases with vascular invasion, suspected malignancy requiring en bloc resection, or tumors larger than 20 cm with limited laparoscopic accessibility. Extensive adhesions from prior surgery and lack of advanced laparoscopic expertise may also serve as relative contraindications. A limitation of our report is the short follow-up period; longer surveillance is needed to properly assess the risk of recurrence.

## Conclusions

Retroperitoneal lipomas are rare and may occasionally present in unusual ways, such as extension through the femoral canal. This can closely mimic more common conditions like a femoral hernia, creating diagnostic and therapeutic challenges. Contrast-enhanced imaging is essential, as it helps define the fat-density lesion and its relationship to nearby structures.

This case highlights the importance of considering a broad differential diagnosis when evaluating groin or thigh masses, particularly in middle-aged women. It also demonstrates that a laparoscopy-assisted hybrid approach can be a safe and effective option for removing such benign tumors, offering the advantages of minimally invasive surgery, precise dissection, and favorable recovery. This technique may serve as a valuable strategy for managing similarly complex tumors while minimizing risk and maintaining patient safety.
